# Correlation between phenotype and polysomnography findings of sleep-disordered breathing in term, non-syndromic infants: a case series study

**DOI:** 10.3389/frsle.2026.1877709

**Published:** 2026-10-19

**Authors:** Liz Lezama, Elisa Basora-Rovira, Aarti Shakkottai

**Affiliations:** Division of Pediatric Pulmonology and Sleep Medicine, University of Texas Southwestern, Children's Health, Dallas, TX, United States

**Keywords:** AHI, airway, apnea, hypoventilation, hypoxemia, infants, laryngomalacia, oxygen

## Abstract

Sleep-disordered breathing (SBD) is understudied in non-syndromic term infants since most of the research focuses on high-risk groups such as premature infants or those with underlying medical conditions. There is a gap in our understanding of phenotypes that predispose this population to experience SDB. Threshold for diagnosis remain poorly standardized, and there is limited data on effective treatment strategies.

**Objective:**

To characterize the clinical presentation and polysomnographic features of term, non-syndromic, infants referred for evaluation of suspected sleep-disordered breathing (SDB).

**Methods:**

This was a single-center case series of review of polysomnographic (PSG) data in non-syndromic term infants from 0–12 months of age from a tertiary care center.

**Results:**

Fifteen infants met inclusion criteria. The mean gestational age was 38.4 ± 0.6 weeks; 80% were male. The mean age at initial PSG was 7.5 ± 3.6 months. Snoring was present in 33% and 47% carried a diagnosis of gastroesophageal reflux (GER). The average apnea-hypopnea index (AHI) was 23.1/h. The mean obstructive AHI (OAHI) was 20/h and the mean central apnea index (CAI) was 3.1/h. Mean oxygen saturation was 97.5 ± 1.5%, with a nadir of 81.0 ± 9.9%. Six patients met criteria for sleep-related hypoxemia (SpO_2_ < 90% for ≥ 5 min). Mean and peak CO_2_ levels were 35 mmHg and 48.6 mmHg, respectively. None of the patients met criteria for sleep-related hypoventilation (CO_2_ > 50 mmHg for > 25% of total sleep time). 71% of the infants underwent an evaluation of their upper airway, and 7 had abnormal findings. Follow-up PSG obtained around 13.4 months, on average, in 10 of the infants showed resolution of SDB in 4 infants. One patient showed resolution of the SDB with CPAP, but was lost to follow-up. SDB was persistent but improved in 5 infants. AHI showed a weak negative correlation with age (ρ = −0.31, *p* = 0.26).

**Conclusion:**

Frequency of moderate-severe obstructive sleep apnea (OSA) is high among non-syndromic term infants. The clinical implication of this is still unclear. However, AHI does appear to decrease with age, and many infants are able to outgrow their OSA without any intervention. Further research is warranted to refine diagnostic thresholds and standardize the diagnostic evaluation and interventions for SDB in this unique population.

## 1 Introduction

Sleep-disordered breathing (SDB) is increasingly recognized as a significant source of morbidity among neonates and infants younger than 6 months of age (Patrinos and Martin, 2017). However, most of the existing research has concentrated on high-risk populations, including premature infants and those with underlying medical conditions Singh et al. (2022). The prevalence of SDB in non-syndromic, full-term infants is estimated in approximately 1 in 1,000 live births; however, its clinical significance remains poorly defined (Patrinos and Martin, 2017; Stefanovski et al., 2022).

In contrast to preterm infants, term infants typically demonstrate a more mature and stable central respiratory network as well as adequate airway and lung development (Patrinos and Martin, 2017). Nevertheless, there are certain physiologic characteristics that make them susceptible to SDB. Specifically, the highly compliant airway of infants predisposes them to dynamic changes in cross-sectional area, whereby small variations in luminal pressure can result in ventilatory instability and obstruction (Horne, 2023). This vulnerability may be compounded by pathophysiologic factors such as paradoxical breathing due to a compliant rib cage, exaggerated laryngeal reflexes, and a predominance of rapid eye movement (REM) sleep (Patrinos and Martin, 2017; Kondamudi et al., 2025; Katz et al., 2012). Congenital abnormalities of the airway, including laryngomalacia, pyriform aperture stenosis, choanal atresia, and laryngeal clefts or webs, further predispose infants to SDB.

Polysomnography (PSG) remains the gold standard for the diagnosis of SDB in infants and children. However, there is limited normative data in infants (Stefanovski et al., 2022; Katz et al., 2012). Moreover, the diagnostic criteria for SDB in infants are identical to that of older children, which generally considers an obstructive AHI > 1 event/hour abnormal, with commonly used severity categories of 1–5 events/hour as mild, 5–10 events/hour moderate, and >10 events/hour as severe; this runs the risk of overestimating the diagnosis in infants (Kombathula et al., 2019). Recent studies have demonstrated that even asymptomatic, healthy infants exhibit a relatively high frequency of obstructive and central apneas during sleep using the current diagnostic thresholds (Stefanovski et al., 2022). Interestingly, many infants with SDB appear to “outgrow” their condition within a few months, even in the absence of any intervention (Stefanovski et al., 2022; Morse et al., 2023).

The paucity of normative data in infants frequently makes clinical decision-making, particularly when considering therapeutic interventions such as continuous positive airway pressure or surgical procedures, challenging. It has also contributed to wide variability in the evaluation, follow-up, and management of SDB in this population (Kondamudi et al., 2025; Stefanovski et al., 2022). The present study aims to characterize the phenotypic and polysomnographic features of a cohort of term, non-syndromic infants evaluated for suspected SDB. Additionally, it seeks to describe follow-up PSG findings, the prevalence of airway abnormalities and gastroesophageal reflux, and the therapeutic interventions administered.

## 2 Methods

This was a single-center case-series of term (≥37 weeks gestational age) non-syndromic infants who were evaluated for symptoms suggestive of SDB and underwent diagnostic PSG between 2017–2023. Participants were identified using an internal registry maintained by the University of Texas Southwestern Medical Center/Children's Health Sleep Disorders Center. Criteria of exclusion included syndromic infants, craniofacial and cardiac abnormalities, preterm infants. All PSGs, whether conducted in the inpatient or outpatient setting, were performed using the standards outlined in the American Academy of Sleep Medicine (AASM) scoring manual. Studies were initially scored by a technician with expertise in scoring pediatric studies and reviewed by a pediatric sleep medicine physician. Electronic medical records were reviewed for demographic data, clinical presentation, results of any further evaluation including flexible and rigid airway endoscopy, and treatment modalities for those diagnosed with SDB. The study was approved by our institutional review board (IRB). Obstructive sleep apnea (OSA) was defined as an apnea-hypopnea index of >2/h, central sleep apnea as apnea-hypopnea index of >5/h, sleep-related hypoxemia as SpO_2_ < 90% > for 5 min, and sleep-related hypoventilation as CO_2_ > 50 mmHg for >25% of total sleep time. Descriptive statistics were used to summarize PSG findings and clinical characteristics. Correlation analyses were performed using the non-parametric Spearman's rank correlation. Statistical significance was defined as a *p*-value < 0.05. Statistical analyses were conducted using Python (version 3.x), data management and preprocessing were conducted using Scipy.

## 3 Results

Fifteen infants met our inclusion criteria. The mean gestational age (GA) was 38.4 +/−0.6 weeks. Majority (80%) of the infants were male (Table 1). Our population was racially and ethnically diverse. Snoring was the most common presenting symptom and was reported in five infants (33%). Figure 1 lists the other complaints that prompted evaluation with PSG. Seven patients (47%) had a history of gastroesophageal reflux (GER). Most of the patients underwent airway evaluation (*n* = 10, 74%) where the most common finding was laryngomalacia (*n* = 4, 40%). Figure 2 lists some of the other airway abnormalities seen in this cohort. Three patients had a normal evaluation.

**Table 1:** Demographics and clinical characteristics of the study cohort (*n* = 15).

Characteristics	Mean ±SD (*n* = 15)
Gestational age	38.4 ± 0.6 (range: 37.0–39.0)
Birth weight	3.2 kg ± 0.40
BMI	14.5 kg/m^2^ ± 6.67
Sex	12 males (80%) 3 females (20%)
Race	White 9 (60%)
	Black 5 (33%)
	Unknown 1 (7%)
Gastroesophageal reflux GER	Yes: 7 (47%) No: 8 (53%)

**Figure 1:**
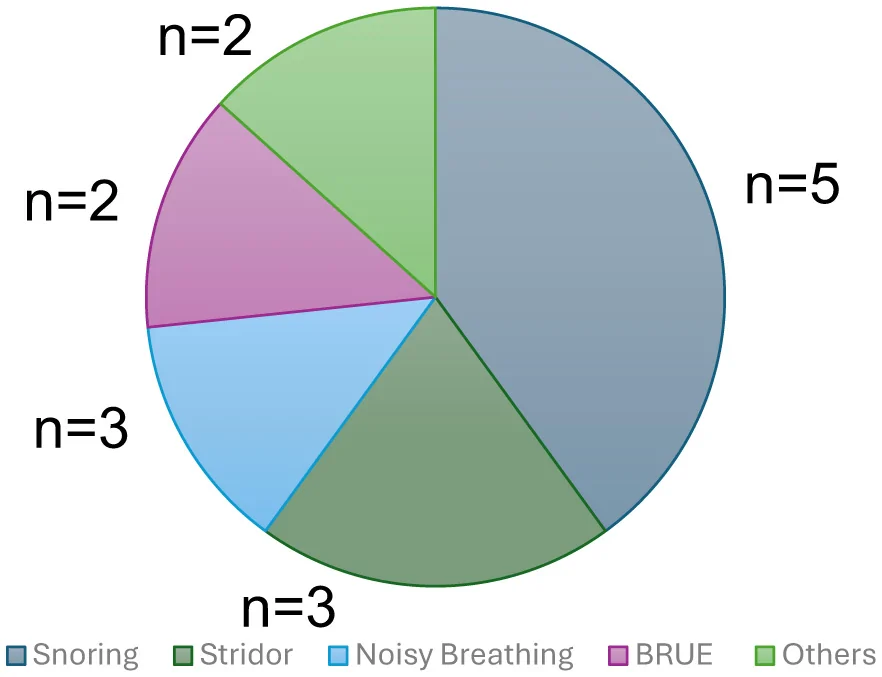
Clinical presentation.

**Figure 2:**
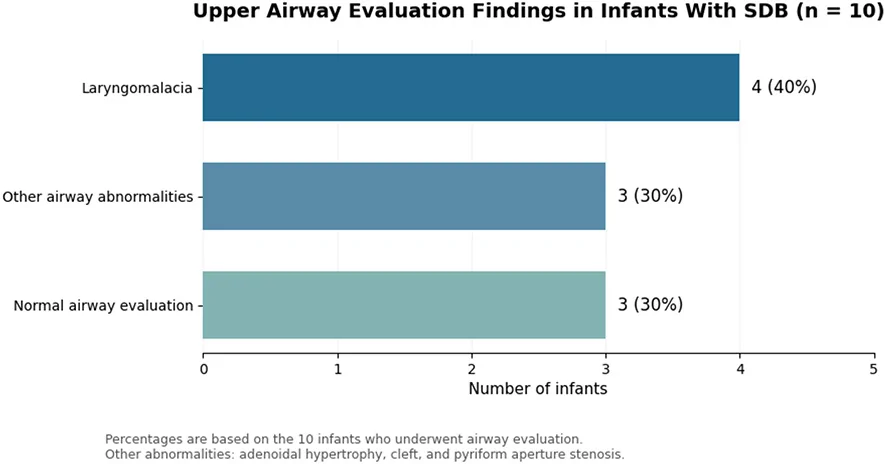
Findings of airway evaluation.

### 3.1 Polysomnography findings

The mean age at initial PSG was 7.5 ± 3.6 months. Participants slept an average of 6.6 h ± 2.2 h, with a sleep efficiency of 78.5 ± 12.5%. The mean arousal index was 15.7 ± 12.0 events/hour. Seven infants (47%) were diagnosed with OSA, defined as an apnea-hypopnea index of >2 events per hour of sleep, calculated for obstructive apneas and hypopneas per hour of sleep during REM and non-REM sleep. Six additional patients (40%) had a diagnosis of both OSA and central sleep apnea (CSA), defined as a central apnea index (CAI)>5/h. Two patients did not have any evidence of SDB on their PSG. Among those with CSA, the longest central apnea was 8.03 s. Periodic breathing was present in 4 patients with an average duration of 2.48 min. The average obstructive AHI (oAHI) was 20.0 ± 19.5 events/hour, with total AHI of 23.1/h, while the CAI was 3.1 ± 3.2 events/hour. Baseline oxygen saturation was 97.5 ± 1.5%, with a nadir of 81.0 ± 9.9%. Six patients met criteria for sleep-related hypoxemia with a mean SpO_2_ < 90% of 15.6 ± 10.9 min. The mean and peak end-tidal CO_2_ levels were 35 mmHg and 48.6 mmHg, respectively. None of the patients met criteria for sleep-related hypoventilation (Table 2). The mean age at follow-up PSG was 13.4 ± 2.3 months. Four out of the 10 patients who underwent repeat PSGs experienced resolution of their SDB, sometime between 24–36 months of age. Five patients had persistent SDB at 19 months of age. Further PSG data was not available for this group to determine if there was resolution of the SDB at an even later age. The one remaining patient showed improvement in the SDB with use of CPAP. However, this patient became lost to follow-up. Therefore, we were unable to get further PSG data or information on duration of PAP use. Correlation analysis revealed a weak negative association between decreasing AHI and age (ρ = −0.31, *p* = 0.26) (Figure 3).

**Table 2:** Polysomnography data (*n* = 15).

Variable	Mean	SD
Age of initial PSG (months)	7.5	3.6
Age of follow-up PSG (months)	13.4	2.3
Total sleep time (TST)	396	129.6
Sleep efficiency (%)	78.5	12.5
Total AHI events/hour	23.1	20.04
Obstructive AHI events/hour	20	19.5
CAI events/hour	3.1	3.2
Respiratory disturbance index (RDI/hr)	22.94	20.17
Arousal index events/hour	15.7	12.0
Periodic breathing minutes	2.48	2.77
Baseline oxygen saturation (%)	97.5	1.5
Duration of sleep-related hypoxemia SpO_2_ < 90% for 5 min	15.6	10.9
Nadir oxygen saturation (%)	81.0	9.9
Mean Co_2_ mmHg	35	15.3
Peak Co_2_ mmHg	48.6	3.1

**Figure 3:**
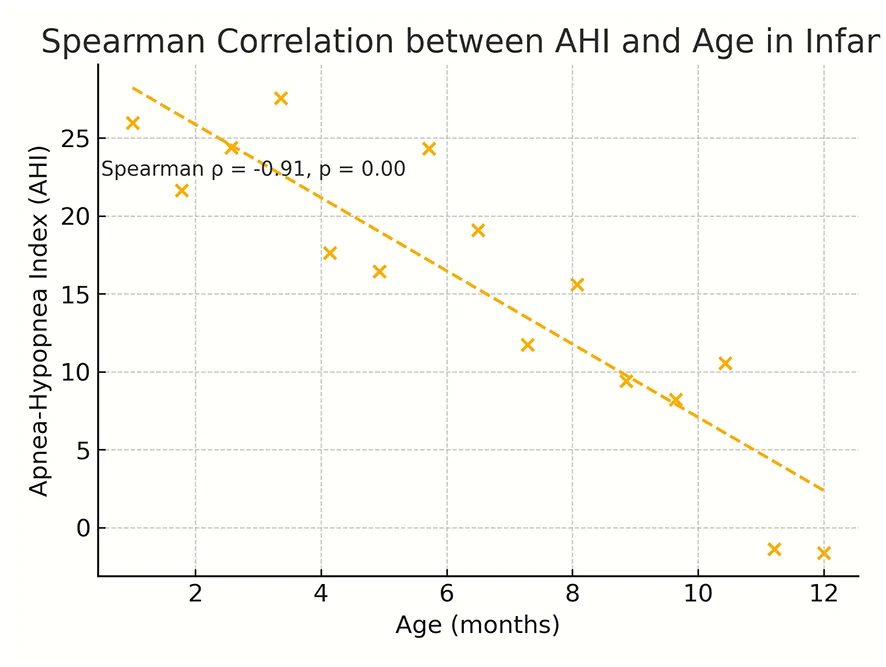
Scatterplot showing the relationship between Apnea-Hypopnea Index (AHI) and age in months among term, otherwise healthy infants. A weak negative Spearman correlation was observed (ρ = −0.31, *p* = 0.26), suggesting a slight decrease in AHI with increasing age.

### 3.2 Treatment modalities

The most common intervention across the cohort was oxygen via low flow nasal canula to a maximum flow of 0.5 L/min. Other treatment modalities that were utilized in our study population included: supraglottoplasty in 3 patients, NIV in the form of CPAP in 2 patients, T&A in 2 patients and 1 patient received high flow nasal canula. We were unable to obtain information on treatment modality in one patient with SDB (Figure 4).

**Figure 4:**
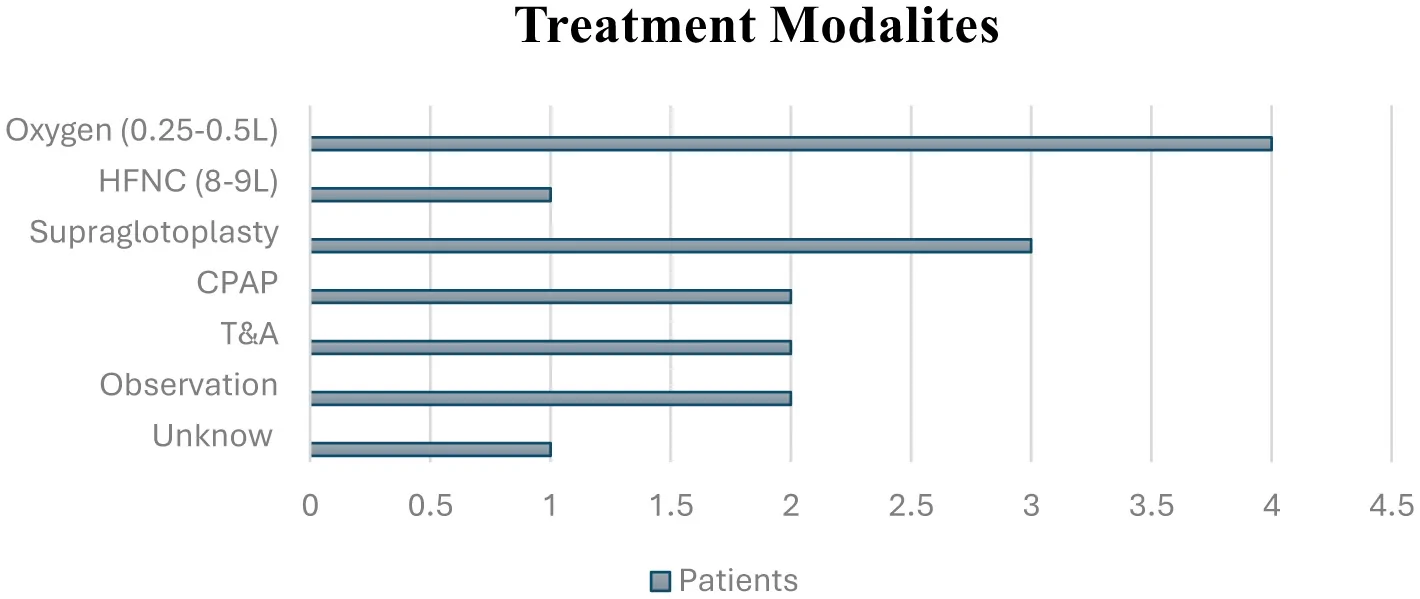
Treatment modalities used in the study cohort.

## 4 Discussion

This was a review of polysomnographic and phenotypic characteristics of term, non-syndromic infants who were evaluated for suspicion of SDB. Although this study was limited by its retrospective design and small sample size, we found some interesting observations. Our cohort had a wide range of symptoms despite being non-syndromic, term, and free of most comorbidities. Obstructive and central sleep apnea were common in this population. At least half of the infants had upper airway abnormalities, such as laryngomalacia and piriform aperture stenosis, that likely predisposed them to have SDB. Almost half of our infants also had GER, which has also been associated with an increased risk for SDB in older children and adults (Stefanovski et al., 2022; Katz et al., 2012). There was a wide variety of treatment modalities offered to our infants with SDB, with oxygen supplementation being the most common. Many of the infants outgrew their SDB at the time of follow-up, suggesting that there is a decrease in AHI with age. We found a weak negative correlation between AHI and age in our limited sample. This is consistent with other studies that have shown similar reduction in AHI over time in infants (Stefanovski et al., 2022; Tapia and Marcus, 2016). Interestingly, not all infants with SDB had a follow-up PSG to ensure resolution of their SDB. Timing of the follow-up PSG also varied between the infants. An important consideration when interpreting our findings is the limited applicability of conventional pediatric OSA severity threshold to infants. Pediatric OSA is commonly categorized according to fixed oAHI threshold; however, respiratory indices undergo substantial developmental changes during the first year of life (Marcus et al., 2012), consequently, application of pediatric threshold may classify respiratory event frequency that occur during normal early development as moderate or severe disease. Our observation of decreasing AHI with increasing age, although limited by sample size and not statistically significant, is consistent with this developmental trajectory. All together, these findings emphasize the need of prospective longitudinal studies in this population to refine age-specific reference values, and importantly, to determine which polysomnographic findings predict outcomes and warrant intervention in this unique population.

## Data Availability

The original contributions presented in the study are included in the article/supplementary material, further inquiries can be directed to the corresponding author.
